# Leveraging Wearable Sensors for the Identification and Prediction of Defensive Pessimism Personality Traits

**DOI:** 10.3390/mi16080906

**Published:** 2025-08-02

**Authors:** You Zhou, Dongfen Li, Bowen Deng, Weiqian Liang

**Affiliations:** College of Computer and Network Security, Chengdu University of Technology, 1#, Dongsanlu, Erxianqiao, Chengdu 610059, China; zhouyou202504@163.com (Y.Z.); 18382616913@163.com (B.D.)

**Keywords:** wearable sensors, temperature sensor, pressure transducer, defensive pessimism, seebeck coefficient, physiological signal, radial artery

## Abstract

Defensive pessimism, an important emotion regulation and motivation strategy, has increasingly attracted scholarly attention in psychology. Recently, sensor-based methods have begun to supplement or replace traditional questionnaire surveys in personality research. However, current approaches for collecting vital signs data face several challenges, including limited monitoring durations, significant data deviations, and susceptibility to external interference. This paper proposes a novel approach using a NiCr/NiSi alloy film temperature sensor, which has a K-type structure and flexible piezoelectric pressure sensor to identify and predict defensive pessimism personality traits. Experimental results indicate that the Seebeck coefficients for K-, T-, and E-type thermocouples are approximately 41 μV/°C, 39 μV/°C, and 57 μV/°C, respectively, which align closely with national standards and exhibit good consistency across multiple experimental groups. Moreover, radial artery frequency experiments demonstrate a strong linear relationship between pulse rate and the intensity of external stimuli, where stronger stimuli correspond to faster pulse rates. Simulation experiments further reveal a high correlation between radial artery pulse frequency and skin temperature, and a regression model based on the physiological sensor data shows a good fit (*p* < 0.05). These findings verify the feasibility of using temperature and flexible piezoelectric pressure sensors to identify and predict defensive pessimism personality characteristics.

## 1. Introduction

Defensive pessimism, an emotion regulation and motivational strategy, has garnered significant attention in the field of psychology [1]. The concept of personality traits, originally introduced by scholars such as Norem, suggests that individuals deliberately envision worst-case scenarios in challenging situations to stimulate motivation, alleviate anxiety, and ultimately enhance performance [2]. In recent years, research on defensive pessimism has expanded beyond basic psychology, influencing cross-cultural studies, educational psychology, and related disciplines, making it a focal point in international academic discussions. For example, at the 2019 Annual Meeting of the Society for Personality and Social Psychology, Nancy Cantor and colleagues explored how defensive pessimism functions as an effective coping mechanism, helping individuals improve performance in uncertain and high-stress situations while regulating emotions and reducing anxiety. Similarly, at the 25th Conference of the Chinese Psychological Society in 2023, Xia Leyi et al. [3] demonstrated that defensive pessimism significantly moderates the relationship between upward social comparison and relative deprivation. These findings highlight the growing importance of studying defensive pessimism in psychological research.

This study integrates sensor network technology to advance research methodologies in defensive pessimism personality assessment. Specifically, it employs temperature and flexible piezoelectric pressure sensors to collect physiological data, offering several advantages over traditional approaches. First, sensors provide real-time physiological and psychological data, mitigating recall bias and the social desirability effect, thereby improving data accuracy and reliability. Second, sensor technology enables long-term, continuous monitoring in natural environments, allowing researchers to capture dynamic emotional fluctuations and behavioral patterns in real-life contexts. Third, real-time data analysis enhances research flexibility, allowing for timely adjustments to experimental strategies and improving research precision.

Traditionally, vital signs have been collected using wired sensor networks. For instance, Hus et al. [4] developed an EEG-based automatic emotion recognition algorithm, achieving classification accuracies of 82.78% for positive/negative valence, 72.91% for high/low arousal, and 61.52% for four-category emotion recognition tasks. Similarly, Hu et al. [5] introduced a PVDF membrane pulse wave sensor for driver drowsiness detection, where power spectral density (PSD) analysis of heart rate sequences revealed that the low-frequency to high-frequency ratio decreased as subjects transitioned from wakefulness to drowsiness. Wang Min et al. [6] proposed a physiological parameter extraction system using a pressure-sensitive cushion, which transmitted real-time pressure data to a computer for physiological state monitoring. In another study, Long Jinyi et al. [7] applied contrastive learning to ECG-based emotion recognition, utilizing data augmentation techniques and a fully convolutional network for feature extraction in a pre-training stage, followed by classification using a multilayer perceptron during fine-tuning. Additionally, Yang Jing [8] designed a wearable inertial sensor system based on a multivariate convolutional neural network (CNN), incorporating adaptive layers and offline training to enable real-time activity recognition through AI analysis.

While these studies present novel approaches for investigating physiological characteristics, several practical challenges remain. Issues such as limited data persistence, low accuracy, high energy consumption, and susceptibility to environmental interference compromise the reliability and consistency of experimental results. Consequently, existing research methodologies fail to comprehensively capture the manifestations of defensive pessimism under diverse conditions, leading to potential misclassification of defensive pessimism as a negative personality trait, which hinders its broader application and understanding.

To address these limitations, this study employs sensor network technology by integrating a thermocouple sensor based on the Seebeck effect and a flexible piezoelectric pressure sensor to measure variations in body surface temperature and radial artery pulse frequency in response to different stimuli. Data processing and analysis are conducted to verify the reliability and consistency of the experimental results. Finally, simulation experiments offer a comprehensive analysis of the relationship between defensive pessimism personality traits and physiological signals, thereby contributing to a deeper understanding of this personality characteristic.

## 2. Principle

### 2.1. Temperature Sensor

**Figure 1:**
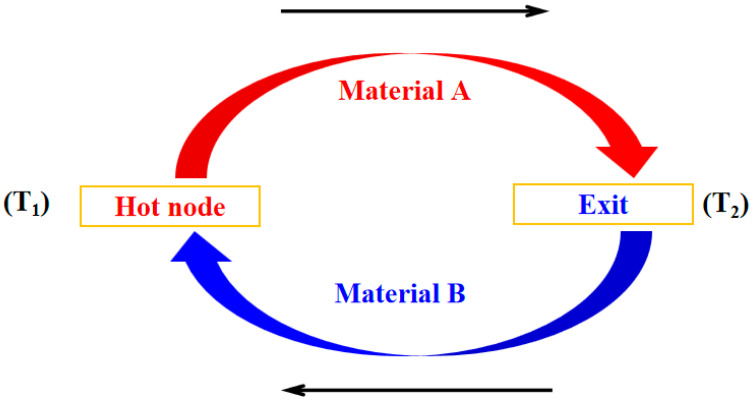
Schematic diagram of the thermoelectric effect.

Thin-film thermocouples are widely employed in high-precision temperature measurement, primarily operating based on the thermoelectric effect [9]. In this experiment, two materials, A and B, with distinct thermoelectric properties, serve as the positive and negative electrodes, forming a closed circuit, as illustrated in Figure 1. When a heat source is applied to the hot junction (T_1_), a temperature gradient is established between the two electrodes. Due to the Seebeck effect, free electrons migrate from the high-temperature region to the low-temperature region, accumulating at the cold junction. This accumulation creates a potential difference between T_1_ and T_2_. In response, the system generates a counteracting current to restore electrostatic equilibrium, ultimately producing a stable thermoelectric electromotive force [10,11].

The calculation Formula (1) of thermoelectromotive force is

E_AB_ = (S_A_ − S_B_) (T_1_ − T_2_) = S_AB_ (T_1_ − T_2_) (1)

In this expression: E_AB_ is electromotive force output by the thin-film thermocouple; T_1_ is the hot end temperature of the thin-film thermocouple; T_2_ is the cold end temperature of the thin-film thermocouple; S_A_ is the Seebeck coefficient of material A; S_B_ is the Seebeck coefficient of material B.

### 2.2. Piezoelectric Voltage Sensor

**Figure 2:**
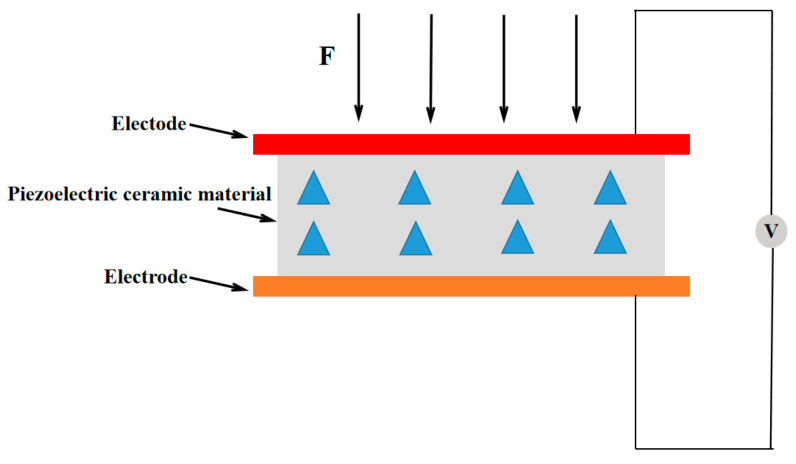
Working principle diagram of piezoelectric sensor.

The key to choosing a pressure sensor is to test the piezoelectric effect of its sensitive materials. Piezoelectric effect means that some materials will generate charges on their surfaces when they receive external force [12]. Similarly, when an electric field is applied, piezoelectric materials will also be deformed. This effect makes the piezoelectric sensor realize the transformation between mechanical signal and electrical signal. This leads to the deformation of the ceramic sheet, which is converted into a charge signal based on the piezoelectric effect. The intensity of this signal is proportional to the value of intensity and velocity. Finally, accurate data are obtained through charge conversion, amplification, filtering, and analog-to-digital conversion [13,14,15]. The schematic diagram is shown in Figure 2 below:

## 3. Design and Preparation of Samples

### 3.1. NiCr/NiSi Alloy Thin-Film Temperature Sensor

NiCr and NiSi alloys are among the most cost-effective metal materials widely used in thermocouple fabrication. These materials exhibit excellent thermal conductivity, with short-term operating temperatures reaching up to 1200 °C and long-term stability maintained at 900 °C [16]. The high nickel content in these alloys enhances their resistance to corrosion and oxidation at elevated temperatures, while also offering high measurement accuracy and rapid response times [16,17]. These advantages make NiCr and NiSi ideal choices for temperature sensor applications that require durability, precision, and high thermal performance.

In this experiment, the temperature sensor is designed using NiCr and NiSi alloys. The fabrication process begins with depositing a 2000 nm alumina layer onto a 0.02 mm thick polyimide substrate, serving as both an insulating and transition layer. Polyimide is selected for its outstanding flexibility, mechanical strength, and electrical insulation properties, making it an ideal substrate for sensors [18]. Alumina is chosen due to its excellent insulating characteristics [19].

**Figure 3:**
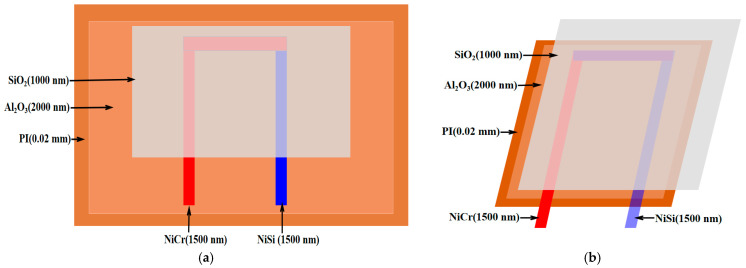
(**a**) Plan view and (**b**) cross-sectional view of the flexible piezoelectric pressure sensor, illustrating its structural layout and multilayer composition. The design includes active piezoelectric layers, encapsulation films, and interconnect electrodes.

**Figure 4:**
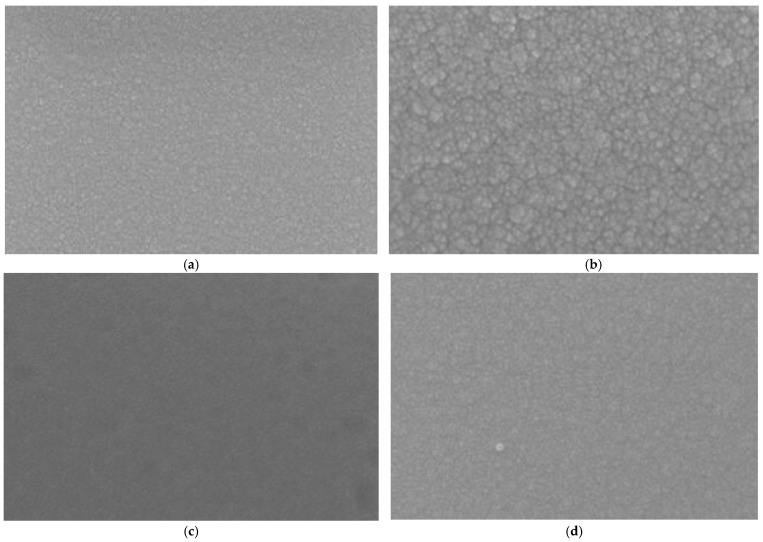
Sem micrograph of NiCr/NiSi alloy thin-film temperature sensor. (**a**) NiCr micrograph; (**b**) NiCr micrograph; (**c**) Al_2_O_3_ micrograph; (**d**) SiO_2_ micrograph.

Next, a 1500 nm NiCr thin film is deposited onto the alumina layer, followed by a 1500 nm NiSi thin film, which functions as the thermal-sensing layer (with NiCr acting as the positive electrode and NiSi as the negative electrode) [20]. To further enhance the stability and oxidation resistance of the NiCr and NiSi thin-film thermocouples, a protective silicon oxide layer is applied to the surface. The example diagram and cross-sectional view of the NiCr/NiSi alloy thin-film temperature sensor are shown in Figure 3 below, and the sem micrograph is shown in Figure 4 below:

### 3.2. Flexible Piezoelectric Pressure Sensor

To prepare the flexible piezoelectric pressure sensor, we first carefully selected the key materials. Specifically, six Cu electrodes with a thickness of 400 nm were fabricated on a polyimide (PI) substrate using electron beam evaporation [21,22]. This technique enables the formation of a uniform and dense metal film with excellent electrical conductivity and structural stability, which is essential for ensuring efficient signal transmission in the sensor.

Subsequently, a zinc oxide (ZnO) thin film with a thickness of 1000 nm was deposited on the surface of each Cu electrode using RF magnetron sputtering [23,24]. This method allows the growth of high-quality ZnO films at relatively low temperatures, providing strong adhesion between the film and the substrate, which is beneficial for enhancing the stability and sensitivity of the sensor. ZnO is selected as the piezoelectric layer due to its high piezoelectric constant and electromechanical coupling coefficient. Moreover, its high resistivity and strong C-axis crystal orientation facilitate efficient conversion of mechanical stimuli into electrical signals, thereby enabling accurate detection of pressure variations [25,26,27].

After ZnO deposition, another 400 nm Cu layer was deposited on top to form a Cu-ZnO-Cu sandwich structure. The top electrode was also fabricated using electron beam evaporation [21,22] to maintain consistency and ensure reliable electrical performance. The resulting multilayer structure combines the excellent conductivity of Cu with the piezoelectric properties of ZnO to achieve optimal device performance.

The Cu-ZnO-Cu structures were then patterned via standard photolithography and wet etching processes to define the required electrode geometry. These patterned structures were integrated onto the PI substrate using conductive adhesive or hot-pressing bonding techniques to ensure mechanical and electrical stability.

Finally, all six composite films were uniformly arranged on a PI substrate measuring 30 mm × 30 mm × 0.02 mm. The layered design of the sensor is carefully engineered to optimize flexibility, sensitivity, and electrical performance for reliable pressure sensing in dynamic environments.

**Figure 5:**
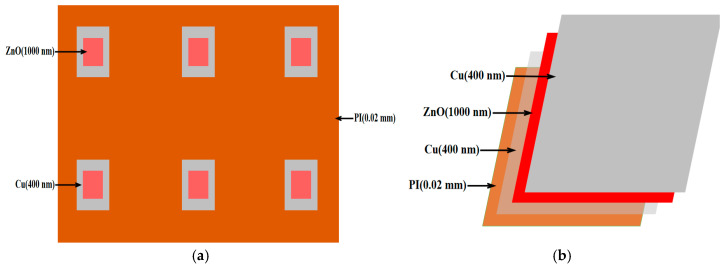
(**a**) Plan view and (**b**) cross-sectional view of the flexible piezoelectric pressure sensor, showing the integration of sensing elements within the device architecture. The cross-sectional view highlights the layer structure and material interfaces.

**Figure 6:**
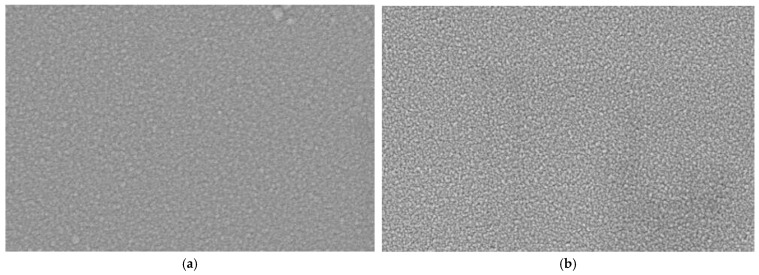
Sem microscopic image of flexible piezoelectric pressure sensor. (**a**) Cu micrograph; (**b**) ZnO micrograph.

Polyimide was chosen as the substrate material due to its excellent flexibility, mechanical robustness, and chemical resistance, making it ideal for wearable and flexible sensor applications [18]. Cu, as the electrode material, not only offers high electrical conductivity but also reduces fabrication costs, ensuring efficient signal collection and transmission [28]. Through this rational selection of materials and layered design, the proposed flexible piezoelectric pressure sensor achieves high-precision pressure detection while maintaining portability and adaptability. The example diagram and cross-sectional view of the flexible piezoelectric pressure sensor are shown in Figure 5 below, and the sem micrograph is shown in Figure 6 below:

## 4. Temperature Static Calibration Experiment

### 4.1. Construction of Experimental Platform

In the temperature static calibration experiment, several pieces of equipment must be prepared in advance, including a constant-temperature oil tank (Suzhou kerun fluid technology co., ltd, Suzhou, China) that provides a stable heat source, an infrared thermometer (Huizhou huicheng district sinaike electronic sales departmentused, Huizhou, China) to measure the temperature of industrial chain oil, a multimeter (Huizhou huicheng district sinaike electronic sales departmentused, Huizhou, China) for voltage measurement, K/T/E-type thermocouples (Nanjing Qinhuai district liuxunze electronic products sales department, Nanjing, China), and a Cu wire(Qinghe county muguiyang trading store, Hebei, China) that connects the thermocouple to the multimeter.

**Figure 7:**
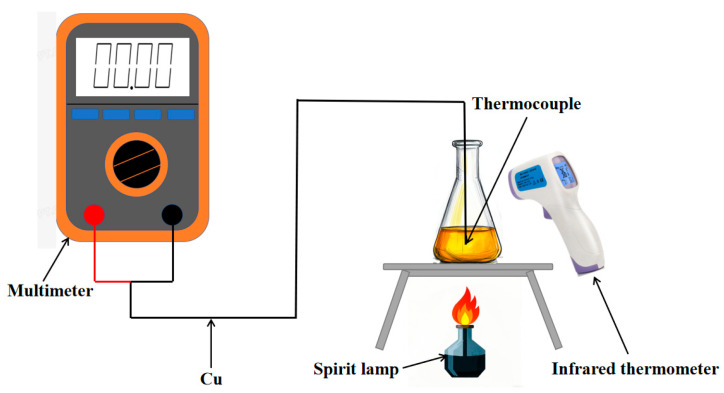
Static calibration experiment platform diagram.

During the experimental setup, a beaker filled with industrial chain oil is placed at the center of an asbestos net. One end of the thermocouple is affixed approximately one-third of the way up the outer wall of the beaker, while the other end is connected to the voltage input of the multimeter via the Cu wire (as illustrated in Figure 7 below).

At the beginning of the experiment, the multimeter is set to the 0–200 mV voltage range by adjusting the function selector knob. The initial voltage reading and the starting temperature of the oil tank are recorded. The alcohol lamp is then ignited to gradually heat the oil. During the heating process, both the multimeter readings and the oil temperature are recorded at 30 s intervals. When the oil temperature stabilizes or the voltage readings no longer change, heating is stopped, and the multimeter is turned off. After the oil cools to room temperature, the experiment is repeated to ensure consistency.

For data analysis, anomalous temperature and voltage readings are first preprocessed. An X–Y scatter plot is then generated, with temperature on the X-axis and voltage on the Y-axis, and the data points are connected. The Seebeck coefficient of the thermocouple is subsequently determined by calculating the slope of the resulting curve.

### 4.2. Static Calibration Result of Thermocouple

In this section, the feasibility and consistency of three types of thermocouples—K, T, and E—are systematically evaluated. The reliability of the experimental data obtained from these thermocouples is then verified.

To minimize experimental errors, two high-precision multimeters are used to measure the Seebeck coefficients of the K-type and E-type thermocouples separately. Additionally, data processing includes outlier elimination and standardization to enhance the accuracy and reliability of the results.

#### 4.2.1. Feasibility Analysis

**Figure 8:**
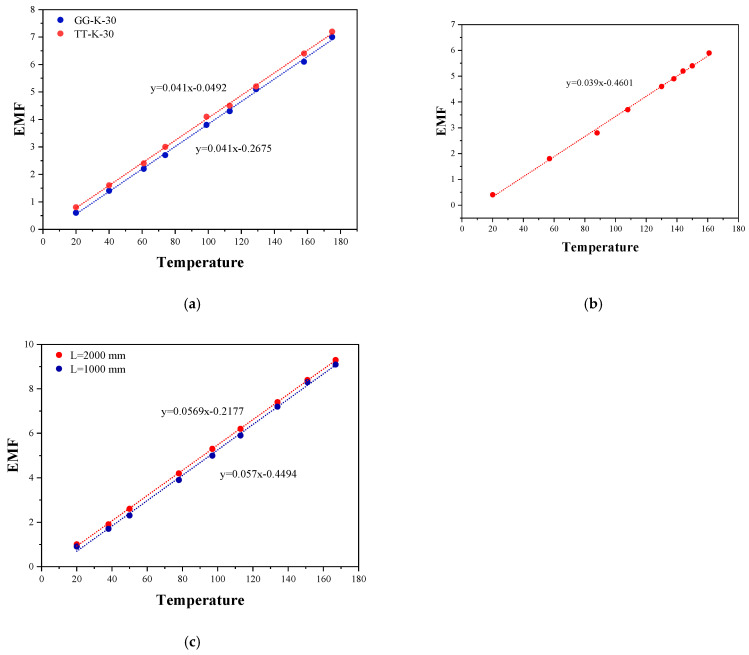
Single-group experimental data diagram of type K/T/E thermocouple. (**a**) K thermocouple; (**b**) T thermocouple; (**c**) E thermocouple.

When measuring the Seebeck coefficient of thermocouple, TT-K-30 (red wire) and GG-K-30 (blue wire) are used for the K-type thermocouple, and L = 1000 mm (red wire) and 2000 mm (blue wire) are used for the E-type thermocouple, respectively. The voltage/temperature function curves are shown in Figure 8 below:

The average voltage/temperature Equation (4) for the K thermocouple is

y = 0.041x − 0.23805(2)

It can be seen from Formula (2) that the average Seebeck coefficient of the two K-type thermocouples is close to 41 μV/°C. Close to the current national standard, the Seebeck coefficient of the K-type thermocouple is 41 μV/°C [29,30]. The voltage/temperature Equation (3) of the T-type thermocouple is experimentally measured as follows:

y = 0.039x − 0.4601(3)

Equation (3) shows that the Seebeck coefficient of the T-type thermocouple is 39 μV/°C. The national standard Seebeck coefficient value of the T-type thermocouple is 39 μV/°C, which shows that the experimental value is the same as the standard value [31]. Finally, the voltage/temperature for two types of E-type thermocouples with different specifications is shown in Equation (4) as follows:

y = 0.05695x − 0.33355(4)

It is easy to know from Equation (4) that the average Seebeck coefficient of E-type thermocouples with two lengths is close to 57 μV/°C, which is close to the current national standard Seebeck coefficient value of 57 μV/°C [32]. To sum up, because the Seebeck coefficients of K-type/T-type/E-type thermocouples are all close to the national standard values, and the maximum error value is within the normal error range, it can be considered that the experimental results have high reliability and accuracy, and the experimental device is available.

#### 4.2.2. Consistency Analysis

##### K-Type Thermocouple

To evaluate the stability and consistency of K-type thermocouples, four sets of experiments were conducted to analyze the voltage–temperature function trends and variations in Seebeck coefficient values. The experiment utilized two types of K-type thermocouples: GG-K-30 and TT-K-30.

**Figure 9:**
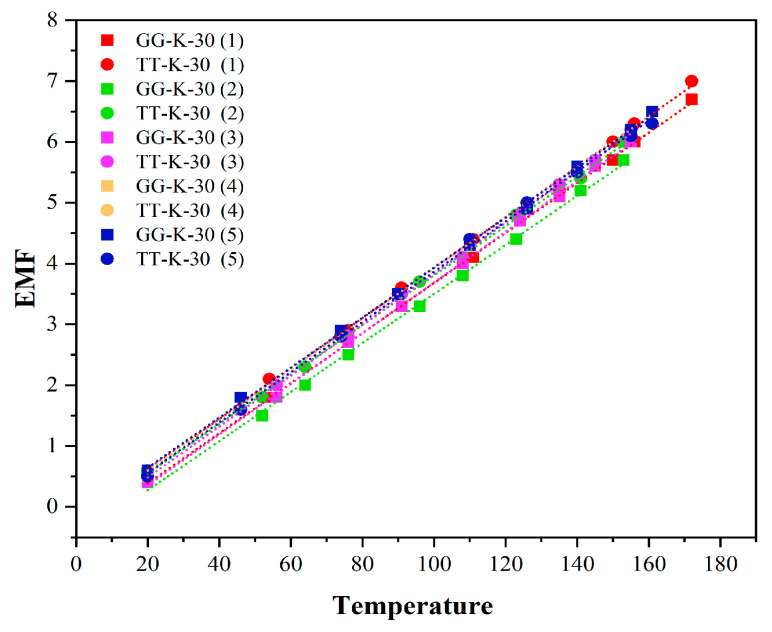
Graph of multiple sets of experimental data for the K-type thermocouple.

Figure 9 presents composite curves of the Seebeck coefficient for the five experimental groups. As shown in the figure, the data points are densely distributed along a straight line, demonstrating a regular distribution pattern. This indicates a high level of consistency across measurements and low data dispersion.

Additionally, the average and variance of the Seebeck coefficients from the five experimental groups were calculated (refer to Table 1 for details). The results show an average Seebeck coefficient of 41.22 μv/°C with a standard deviation of 0.4826. This average value closely aligns with the national standard for K-type thermocouples (41 μv/°C), confirming the high accuracy and reliability of the collected data for further research [29,30]. The small standard deviation further suggests minimal fluctuation in the measured Seebeck coefficients, reinforcing the low dispersion of the data.

The experimental results presented in Figure 9 and Table 1 strongly support the reliability of the study’s conclusions. These findings systematically validate the measurement performance of K-type thermocouples while establishing a multi-dimensional verification system, providing robust empirical evidence for the consistency and stability of K-type thermocouple data.

##### T-Type Thermocouple

**Figure 10:**
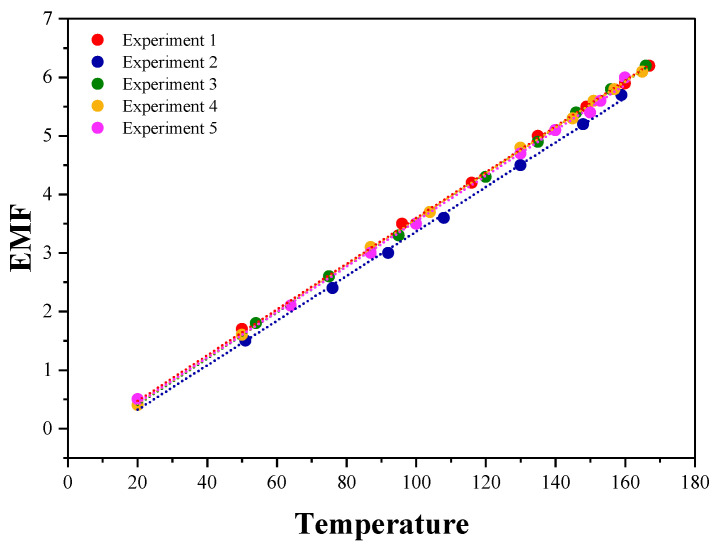
Graph of multiple sets of experimental data for the T-type thermocouple.

To verify the consistency and reliability of the Seebeck coefficient for T-type thermocouples, five sets of measurements were conducted. Figure 10 presents the voltage–temperature function curves for these experiments, clearly showing that all data points are closely aligned along a linear trend, indicating a high degree of linear correlation. This pattern suggests minimal variation between experimental results, low data dispersion, and strong measurement consistency.

Table 2 summarizes the Seebeck coefficient values obtained from the five experimental groups, yielding an average value of 38.98 μV/°C with a standard deviation of 0.5404. Since the standard deviation is small, it confirms that the data distribution is concentrated, further reinforcing the consistency of the measurements and the reliability of the experimental results. Additionally, the national standard Seebeck coefficient for T-type thermocouples is 39 μV/°C, which closely aligns with the experimentally obtained average, with the deviation falling within the acceptable tolerance range [31].

In conclusion, both the data distribution observed in Figure 10 and the statistical results in Table 2 validate the accuracy and reliability of the experimental measurements. The strong linear correlation among multiple test groups provides robust empirical support and a solid scientific foundation for further research on T-type thermocouples.

##### E-Type Thermocouple

**Figure 11:**
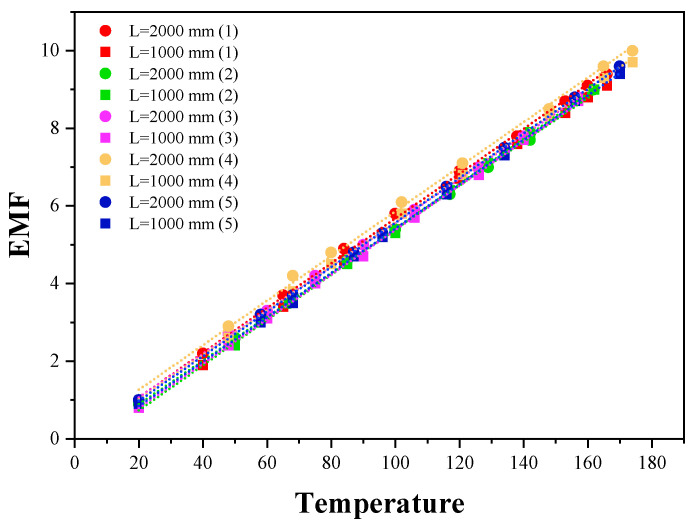
Graph of multiple sets of experimental data for the E-type thermocouple.

To verify the consistency of the Seebeck coefficients of E-type thermocouples, five sets of tests were conducted using two types of E-type thermocouples with different lengths. Figure 11 presents the composite voltage/temperature curves from these five experiments, resulting in a total of ten sets of Seebeck coefficient values for the E-type thermocouples. As shown in the figure, the data points closely align with the central linear line, demonstrating a compact distribution and a strong linear trend. This indicates that the dispersion of the experimental data is minimal, reflecting high data consistency.

Table 3 provides the specific Seebeck coefficient values for the five sets of data, followed by the calculation of the average value (57.23 μv/°C) and the standard deviation (0.5618). The small standard deviation further confirms the low data dispersion, indicating that the experimental results are stable and that the consistency across multiple sets of experiments is high, with minimal variation. The average values from the five experiments also align closely with the national standard Seebeck coefficient for E-type thermocouples, which is 57 μv/°C [32]. This demonstrates that the Seebeck coefficient characteristics of the E-type thermocouple meet the performance standards set by the national specifications, making them suitable for subsequent research.

In conclusion, both the data distribution shown in Figure 11 and the statistical results in Table 3 confirm that the measurements of the E-type thermocouple in this experiment are reliable. The high consistency between multiple experimental results provides a solid foundation for the future application of E-type thermocouples.

## 5. Radial Artery Frequency Test Experiment

### 5.1. Performance Test of Flexible Piezoelectric Pressure Sensor

Before conducting the actual measurement of the radial artery pulse frequency, we performed a series of preliminary experiments to evaluate the sensitivity and response performance of the flexible piezoelectric voltage sensor. Specifically, we carried out four validation tests: (1) dynamic human test data, (2) stability under pulsed cyclic loading, (3) long-term wearability test, and (4) environmental adaptability verification. These tests were designed to ensure the sensor’s reliable performance under repeated use and to demonstrate its strong potential for wearable health monitoring. The experimental results are summarized in Table 4, Table 5, Table 6 and Table 7:

In conclusion, the sensor demonstrated reliable sensitivity and response to human pulse signals, sustained performance over multiple loading cycles, stable output during prolonged wearing conditions, and minimal degradation under varying ambient environments [33,34]. These results confirm the sensor’s robustness and feasibility for wearable health monitoring applications, particularly in tracking radial artery pulse under dynamic conditions.

### 5.2. Experimental Results of Radial Artery Pulsation Frequency Test

**Figure 12:**
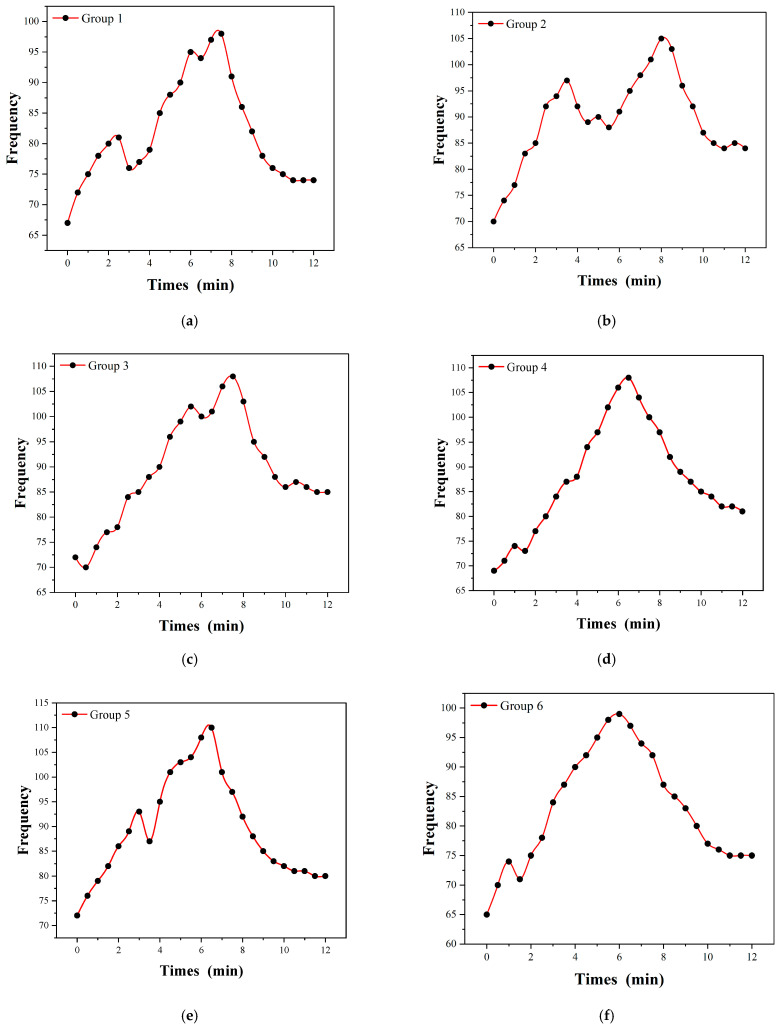
Radial artery pulse responses of six healthy human subjects under identical external stimuli. Subfigures (**a**–**f**) correspond to Subjects 1 through 6, respectively. Each subfigure illustrates the time-domain waveform of the radial artery pulsation as recorded by the flexible piezoelectric pressure sensor. The pulse waveforms exhibit a high degree of consistency across different individuals, indicating that the physiological responses to the stimuli are stable and repeatable. (**a**) Group 1; (**b**) Group 2; (**c**) Group 3; (**d**) Group 4; (**e**) Group 5; (**f**) Group 6.

Figure 12 shows the test results of radial artery pulsation frequency over time, as measured by flexible piezoelectric pressure sensors, in six groups of college student subjects (aged approximately 20 years). All subjects underwent the experiment under identical external stimulation conditions. The stimulation consisted of viewing a carefully selected emotional video with a family-related theme designed to elicit natural emotional responses. The video content was the same for all subjects, with consistent duration, narrative structure, and emotional progression. Specifically, the emotional stimulus was delivered through a 0–9.5 min video that portrayed emotionally engaging family scenarios, which could trigger varying degrees of emotional arousal depending on the subject’s personality traits. These individualized emotional responses were expected to manifest physiologically, particularly in the pulsation frequency of the radial artery. Although the formal stimulation period was 0–9.5 min, the recording was extended to 12 min to include the subject’s physiological recovery phase.

The experiment was conducted sequentially for all six groups under identical environmental conditions. During the process, the pressure sensor was fixed to the subject’s wrist above the radial artery, and an oscilloscope was used to continuously monitor and record the pulsation signals. The resulting frequency–time waveforms for each group are shown in Figure 12, capturing both the response to the stimulus and the post-stimulus return to baseline:

From Figure 12a, the pulse frequency of radial artery gradually increased in the initial period (0–4 min), from about 70 beats/min to about 90 beats/min. Then it reached the peak (about 100 beats/min) in the 5th to 7th min, before showing an obvious decrease until it finally stabilized. This shows that the subjects have obvious physiological stress response after stimulation, but they recover quickly.

Figure 12b shows that the pulse frequency increased twice during the whole test stage. The first peak was at about the 4th min (about 95 beats/min), decreased briefly, then rose to the second peak near the 8th min (about 105 beats/min) before finally falling back slowly. It shows that the subject may have a delayed response or multi-stage response to external stimuli.

Figure 12c reveals that the pulse frequency gradually increased from the initial 70 beats/min to the peak near the seventh minute (about 110 beats/min) and then decreased rapidly. The overall response of the subject is “single and clear”, showing a typical “excitement-recovery” fluctuation trend.

Figure 12d shows that the pulse frequency rose more gently, rising from about 70 beats/min to the peak at about 6 min (about 110 beats/min), and then slowly falling towards the end. The data curve is more “symmetrical” than other groups, which shows that the autonomic nervous response of the subject is more balanced and the adaptation process is mild.

Figure 12e indicates that the pulse frequency showed a smooth and steady increase during the initial 6.5 min, starting at 72 beats/min and gradually peaking at 110 beats/min. This progression indicates a consistent rise in arterial activity, potentially reflecting a stable physiological response to external stimuli. Notably, a slight fluctuation was observed around the 3.5 min mark, where the value temporarily dipped to 87 beats/min before rising again, suggesting a brief adjustment phase. After reaching its peak, the pulse frequency began a moderate decline, ending at 82 beats/min by the 10 min mark. The curve overall presents a balanced transition with identifiable stages of rising and falling.

Figure 12f exhibited a more fluctuating pattern in the early stages, starting from a lower baseline of 65 beats/min and rising unevenly. A brief stagnation is visible between 1.5 and 2.5 min, followed by a sudden surge reaching 95 beats/min at the 5 min point. The mid-phase values (5–6.5 min) remained relatively high, indicating a strong physiological response, before gradually tapering off toward the end. By the 10 min mark, the pulse frequency had decreased to 77 beats/min. The overall trend reflects a delayed but strong response followed by a progressive decline.

### 5.3. Experimental Data Analysis of Radial Artery Pulse Frequency Test

For the first 0–3 min, emotionally stimulating videos were presented to the subjects, during which the pulse frequency of the radial artery increased rapidly. This initial rise is attributed to the acute activation of the sympathetic nervous system in response to the stressor, a typical physiological reaction known as the “fight or flight” response [35].

Between approximately 3.5 and 4.5 min, the rate of increase slowed down significantly, and in some subjects, a slight decline in heart rate was observed. This phase is likely due to short-term physiological adaptation or habituation, in which the neuroendocrine system begins to moderate its response to continuous stimulation, as documented in previous studies [36].

From 4.5 min to around 7 min, the pulse frequency rose again, eventually reaching its peak. This secondary increase may be related to the emotional climax induced by the video content, as emotional arousal often fluctuates in waves during prolonged exposure, which is also closely related to the inhibition and recovery of sympathetic–parasympathetic nerve regulation [37,38].

After approximately 7 min, the pulse frequency of the radial artery began to decline. This decrease was primarily caused by the reduction in external emotional stimuli, as the video entered its final, calming phase. During this stage, participants’ emotional states gradually returned to a more relaxed condition [39]. By the end of the video (around 9.5 min), the subjects were re-exposed to a neutral environment, resulting in a further stabilization of heart rate toward baseline levels. This physiological response reflects the disengagement from emotional arousal and the onset of recovery, driven by the withdrawal of external stimuli and the reactivation of parasympathetic nervous activity [40].

These temporal changes in heart rate dynamics support the feasibility of using our flexible piezoelectric pressure sensor to monitor pulse frequency under emotional stimulation. Compared with traditional pressure sensors, our flexible voltage-type sensor offers higher accuracy, reduced environmental interference, and greater stability in waveform acquisition, thereby ensuring continuous and reliable data collection [41].

To sum up, the six groups of experimental data demonstrate that the radial artery pulse rate generally increases during exposure to external emotional stimuli and gradually returns to baseline afterward, with only minor fluctuations during the stimulation period. This trend, consistently observed across subjects, indicates that the frequency–time response curve effectively reflects real-time physiological changes triggered by emotional input. In this study, the frequency–time curve of radial artery pulsation, as presented in Figure 12, serves as the primary feature for analysis and prediction. These curves were derived from continuous waveform recordings collected by flexible piezoelectric pressure sensors, ensuring high temporal resolution and minimal environmental interference.

To assess the consistency of physiological responses among the six subject groups, we calculated the average Pearson correlation coefficient between their frequency–time curves, resulting in a value of 0.823557. This high correlation confirms both the stability of the sensor measurements and the similarity in response trends under identical stimulation. However, despite this overall consistency, subtle differences in the pulsation patterns were also observed—particularly in the timing and amplitude of pulse rate changes—which may be attributed to individual differences in personality traits.

According to previous studies, individuals with different personality characteristics exhibit distinct physiological reactions to the same emotional stimuli [42]. Therefore, the nuanced variations in these frequency–time curves can serve as preliminary features for identifying defensive pessimism traits. Compared with traditional measurement tools such as stethoscopes, the flexible piezoelectric sensor-based method used in this experiment offers superior accuracy, continuity, and robustness, making it more suitable for capturing fine-grained physiological data relevant to psychological state analysis. These findings collectively validate both the reliability of the data and the potential of the proposed approach for personality-related prediction tasks.

## 6. Simulation Experiment

To verify the effectiveness of the temperature sensor and the flexible piezoelectric pressure sensor in identifying and predicting defensive pessimism personality characteristics, this section designs and conducts three sets of simulation experiments:

### 6.1. Correlation Simulation and Correlation Analysis of Physiological Indexes

In the correlation simulation and analysis of physiological indexes, a time axis (T) is first set with a uniform distribution from 0 to 10 min, which serves as the time dimension for the data. Then, based on the pulse frequency of the radial artery, conditional expressions are established for different time intervals to fit the experimental data, allowing for the calculation of pulse_rate. For the generation of skin temperature data, the reference ambient temperature is set first, followed by the calculation of temperature variation (temp_variation) based on changes in the radial artery pulse frequency. The final skin temperature (temperature) is then determined.

**Figure 13:**
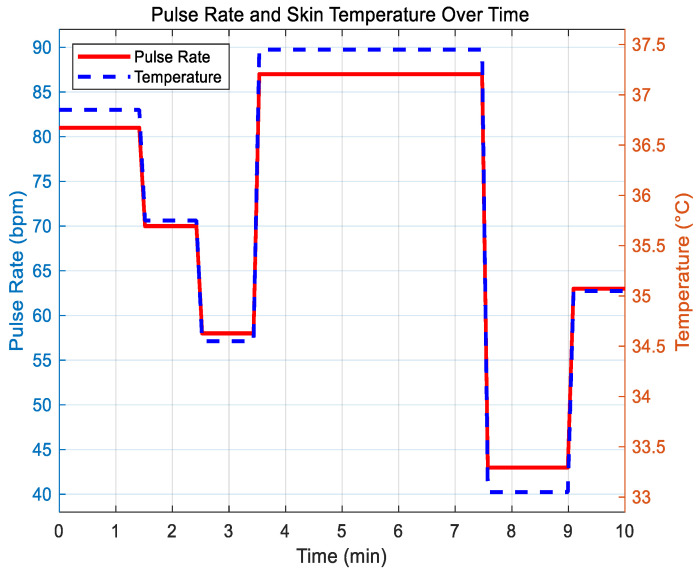
Correlation simulation results of physiological indicators.

Using Matlab, draw a bivariate function, and visually display the curve of radial artery pulsation and skin temperature with time in a graph (Figure 13 below). Calculate the correlation coefficient between these two variables to evaluate their correlation degree.

From Figure 13, it can be observed that the pulse frequency of the radial artery fluctuates significantly at different time intervals, simulating the physiological response changes oin the human body under varying external stimuli. The skin temperature also exhibits similar fluctuations over time, showing a high correlation with the radial artery pulse frequency. The correlation coefficient between the two variables is close to 1, indicating a strong correlation between them. This suggests that skin temperature will change correspondingly when the pulse frequency of the radial artery changes in the set model.

In this simulation experiment, the signals measured by the temperature sensor and flexible piezoelectric pressure sensor demonstrate a certain level of correlation and predictable change patterns. Given that defensive pessimism may alter the physiological state of the human body, these changes can be reflected in temperature and radial artery pulsation [42,43]. Therefore, based on the correlation and measurability of the physiological indexes in the simulation results, it is shown that the temperature sensor and flexible piezoelectric pressure sensor can theoretically capture physiological signal changes related to defensive pessimism personality traits, which can then be used to identify and predict these characteristics.

### 6.2. Correlation Simulation and Filtering Optimization Analysis of Radial Artery Pulse Frequency and Skin Temperature

In the section exploring the correlation simulation and filtering optimization analysis between radial artery pulsation frequency and skin temperature, the initial dataset is constructed by inputting the set time points and corresponding radial artery pulsation frequencies. Next, a fourth-order polynomial regression is applied to fit the pulse frequency data of the radial artery, generating a continuous frequency change curve. Based on the principle of the K-type thermocouple, the fitted pulse frequency and skin temperature data are processed to reduce noise interference. The out-of-bag (OOB) error between the original fitted radial artery pulse frequency and the filtered data is then calculated to evaluate the effectiveness of the filtering process. Subsequently, the original fitting curve and the filtered curve are plotted on the X–Y graph for visual comparison. Finally, the correlation coefficient between the filtered radial artery frequency and skin temperature is calculated to analyze their correlation.

**Figure 14:**
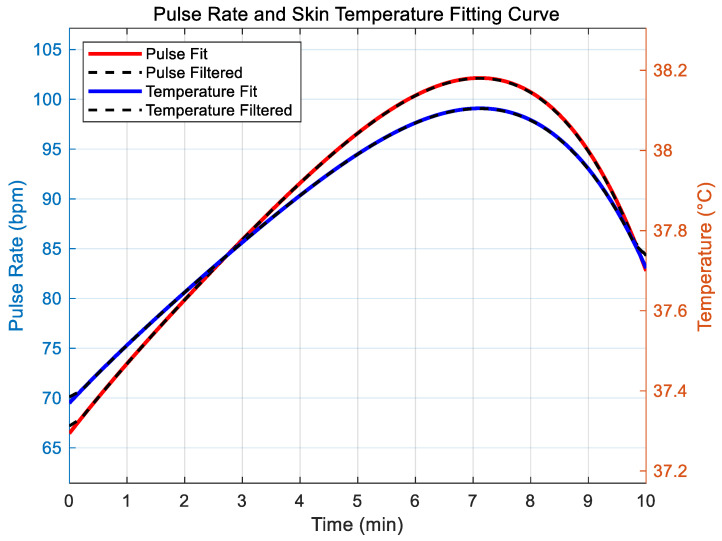
Correlation simulation and filtering optimization analysis of radial artery pulse frequency and skin temperature.

From Figure 14, it can be concluded that the fourth-order polynomial fitting effectively captures the data’s changing trend for radial artery pulsation frequency. The filtered curve is smoother than the original, indicating that the moving average filtering successfully reduces data fluctuation and noise interference. The OOB error result quantitatively reflects the degree of data optimization achieved through filtering. The graph also shows a strong linear correlation between skin temperature and radial artery pulse frequency, consistent with the temperature model based on the K-type thermocouple principle. The consistency of the two curves further highlights the close relationship between radial artery pulse frequency and skin temperature.

In this experiment, by simulating radial artery pulse frequency and skin temperature data and conducting a series of data processing and analysis, it was found that there is a high correlation between the two. Defensive pessimism personality traits may cause changes in the physiological state of individuals under external stimuli, which can lead to alterations in physiological indexes such as radial artery pulse frequency and skin temperature. Temperature sensors and sensors for measuring radial artery pulsation (such as the flexible piezoelectric pressure sensor used in this study) can capture these physiological changes. Therefore, based on the simulation results, these sensors have the potential to detect physiological signal changes related to defensive pessimism personality traits and can be used to identify and predict this personality trait.

### 6.3. Research on Data Feature Extraction and Personality Recognition Modeling of Physiological Sensor

**Figure 15:**
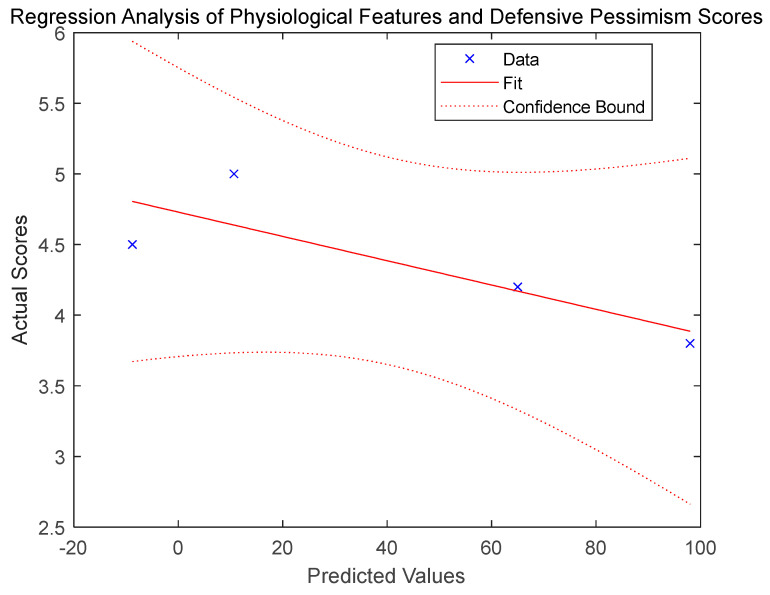
Regression analysis of physiological characteristics and defensive pessimism score.

**Figure 16:**
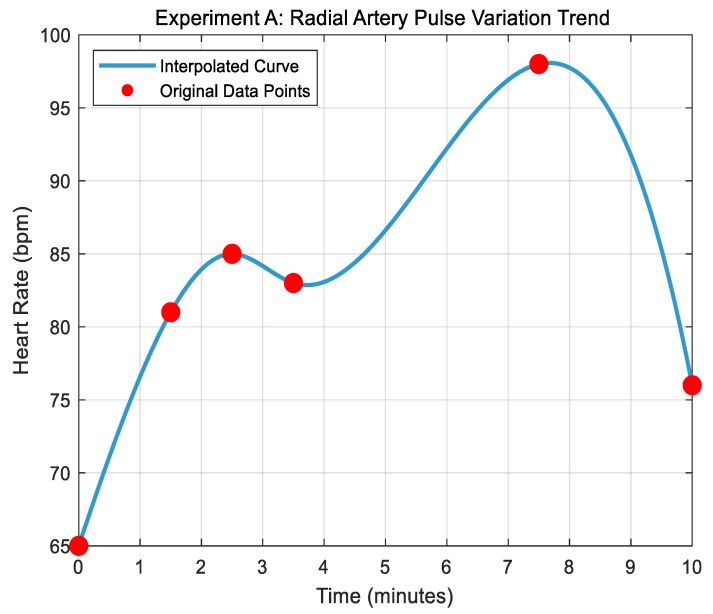
Trend diagram of radial artery pulsation in Experiment A.

This section focuses on feature extraction from experimental data measured by two types of sensors and the modeling of personality characteristic recognition. First, the Seebeck coefficient data of the K/T/E thermocouple is imported, and the reliability of the experimental results is validated through data analysis of the thermocouple’s static calibration experiment. Next, the radial artery pulsation data from Experiment A (the first experiment in the radial artery pulsation frequency study) is processed. Time nodes and corresponding heart rates are defined, and a continuous heart rate curve within a 0–10 min time frame is generated using spline interpolation (as shown in Figure 15 below). From this, key data values such as the initial heart rate, peak value, and rate of increase are extracted. Assuming a defensive pessimism score, the extracted physiological characteristics are combined into a feature matrix, and a multiple linear regression model (As shown in Figure 16 below) is constructed to explore the relationship between physiological characteristics and the defensive pessimism score, with the results visualized. Finally, the experimental heart rate curve is plotted, visually displaying both the interpolation curve and the original data points, and key conclusions, such as measurement system verification, physiological characteristic analysis, and regression model results, are provided.

In this simulation experiment, the heart rate curve of Experiment A showed a pattern of a rapid rise followed by a steep drop, with a peak value of 98 beats per minute. This pattern aligns with expectations under defensive pessimism stress, indicating that physiological characteristics can effectively reflect stress states. Furthermore, the adjusted R-squared value of the regression model demonstrates a good fit, and the *p*-value is less than 0.05, showing a significant correlation between physiological characteristics and the defensive pessimism score. This means that physiological characteristics can explain, to some extent, changes in the defensive pessimism score, providing strong support for identifying related personality traits based on physiological sensor data.

In summary, the simulation results confirm the reliability of the system. Additionally, a significant correlation is found between the physiological characteristics of radial artery pulsation and the defensive pessimism score. This implies that, in practical applications, temperature sensors and flexible film piezoelectric pressure sensors can capture the changes in physiological signals related to defensive pessimism personality traits, which can be used to identify and predict these personality characteristics.

## 7. Conclusions

This paper focuses on the identification and prediction of defensive pessimism personality characteristics, proposing an innovative and efficient testing method based on sensors. In terms of the temperature sensor, the Seebeck coefficient of the K/T/E thermocouple is found to be close to the national standard. Specifically, the average Seebeck coefficient of the K thermocouple is 41.22 μv/°C, with a standard deviation of 0.4826. The average value of the T thermocouple is 38.98 μv/°C, with a standard deviation of 0.5404, while the average value of the E-type thermocouple is 57.23 μv/°C, with a standard deviation of 0.5618. These results ensure the reliable performance of the sensor.

In the radial artery frequency test experiment, all six groups of experimental data show that the radial artery pulse rate is highly correlated with the intensity of external stimuli. With the application of external stimuli, the radial artery pulse typically exhibits a trend of first rising and then falling, reflecting both the feasibility of the experimental data and the reliability and stability of the experimental instruments.

Furthermore, this study further validates the feasibility of using the system to predict defensive pessimism personality characteristics through simulation experiments. The correlation simulation and analysis of physiological indexes demonstrate that the pulse frequency of the radial artery is highly correlated with the trend of skin temperature changes, with a correlation coefficient close to 1. The correlation simulation and filtering optimization analysis of radial artery pulse frequency and skin temperature show that data fluctuations are reduced after filtering, and the relationship between them is highly linear. The regression model built from the feature extraction of physiological sensor data and personality recognition modeling shows a good adjusted R-squared value, with a *p*-value less than 0.05, indicating a significant correlation between physiological characteristics and defensive pessimism scores.

In conclusion, this study demonstrates that NiCr/NiSi alloy film temperature sensors and flexible piezoelectric pressure sensors can effectively capture changes in physiological signals related to defensive pessimism personality characteristics, both theoretically and experimentally. This provides a novel method and foundation for the identification and prediction of defensive pessimism personality traits.

## Figures and Tables

**Table 1:** K-type thermocouple multi-group experimental data analysis table.

Group	Seebeck Coefficient (μV/°C)	Average (μV/°C)	Standard Deviation
1	41.2	41.27	0.473872932
	41.5		
2	40.3		
	40.7		
3	41.7		
	41.9		
4	41.5		
	41.4		
5	41.1		
	41.4		

**Table 2:** T-type thermocouple multi-group experimental data analysis table.

Group	Seebeck Coefficient (μV/°C)	Average (μV/°C)	Standard Deviation
1	39.1	38.98	0.540370243
2	38.1		
3	39.5		
4	39.3		
5	38.9		

**Table 3:** E-type thermocouple multi-group experimental data analysis table.

Group	Seebeck Coefficient (μV/°C)	Average (μV/°C)	Standard Deviation
1	57.7	57.23	0.561842208
	57.2		
2	56.4		
	58.3		
3	56.4		
	57.3		
4	57.4		
	57.1		
5	57.4		
	57.1		

**Table 4:** Dynamic human test data (*n* = Five healthy volunteers).

Test Scenario	Key Parameter	Sensor Output Stability (CV%)	Fidelity of Characteristic Waveform	Motion Jamming Signal-to-Noise Ratio (dB)
Resting state (5 min)	Heart rate: 68 ± 3 BPM	1.2%	P_1_–P_5_ Full feature detection	N/A (No movement)
Wrist 90° flexion and extension	Flexion and extension frequency: 0.5 Hz	8.7% (Action period)	P_1_/P_4_ Amplitude retention > 95%	24.5
Moving state (4 km/h)	Acceleration interference: 0.3–2 g	4.3%	P_3_ Characteristic ambiguity < 10%	19.8
Language communication	Interference of vocal cord vibration conduction	3.1%	No additional impurity peak	28.1
Deep breathing (0.1 Hz)	Chest pressure fluctuation amplitude: ±2 kPa	6.5%	Baseline drift <5%	17.2

In Table 4: CV%: Coefficient of variation in current output. dB = pulse signal RMS/interference signal RMS.

**Table 5:** Stability of pulsed cyclic loading (simulating arterial pulsation).

Test Condition	Cycles	Sensitivity Attenuation (S_1_/S_0_)	Baseline Drift (ΔI/I_0_)	Response Time
10 kPa, 1 Hz (Resting simulation)	20,000	−2.1%	+0.8%	15 ms→16 ms
40 kPa, 1.8 Hz (Motion simulation)	20,000	−5.3%	+3.2%	15 ms→18 ms
Impact load (100 kPa, 0.5 s)	5000	−1.7%	+1.1%	No change

Test method in Table 5: The radial artery pressure waveform was simulated using a pneumatic piston, characterized by a rising edge of 20 ms and a plateau phase of 200 ms. Data were acquired at a sampling frequency of 1 kHz.

**Table 6:** Stability of pulsed cyclic loading.

Time Interval	Heart Rate Error (BPM)	Waveform Coefficient K Drift	Signal Attenuation (ΔA/A_0_)	Environmental Disturbance Event
0–4 h	0.3 ± 0.2	0.002	−0.5%	Room temperature meditation
4–8 h	0.8 ± 0.5	0.005	−1.2%	Eating (increased hand activity)
8–12 h	1.2 ± 0.7	0.008	−2.1%	Walk for 30 min
12–24 h	2.1 ± 1.0	0.012	−3.8%	Sleep posture change

In Table 6: Waveform coefficient K = (P_m_ − P_5_)/(P_1_ − P_5_).

**Table 7:** Environmental adaptability verification.

Interference Source	Abnormal Sensor Output	Recovery Time	Clinical Parameter Error
Sweat soaking (NaCl 0.9%)	Instantaneous noise + 15% (Lasts for 10 s)	<30 s	Heart rate + 0.8 BPM
Sudden temperature change (25→40 °C)	Baseline migration + 7.3%	120 s	K value + 0.006
Electromagnetic interference (GSM 1.8 GHz)	No abnormality was detected.	N/A	No influence
Lateral shear force (30°)	P_3_ amplitude reduction 12%	Recovers immediately	DAI influence + 3%

## Data Availability

The original contributions presented in this study are included in the article. Further inquiries can be directed to the corresponding authors.
